# Increased *SOX2* Gene Copy Number Is Associated with *FGFR1* and *PIK3CA* Gene Gain in Non-Small Cell Lung Cancer and Predicts Improved Survival in Early Stage Disease

**DOI:** 10.1371/journal.pone.0095303

**Published:** 2014-04-15

**Authors:** Luca Toschi, Giovanna Finocchiaro, Teresa T. Nguyen, Margaret C. Skokan, Laura Giordano, Letizia Gianoncelli, Matteo Perrino, Licia Siracusano, Luca Di Tommaso, Maurizio Infante, Marco Alloisio, Massimo Roncalli, Marta Scorsetti, Pasi A. Jänne, Armando Santoro, Marileila Varella-Garcia

**Affiliations:** 1 Division of Oncology and Hematology, Humanitas Clinical and Research Center, Rozzano, Milan, Italy; 2 University of Colorado School of Medicine, Aurora, Colorado, United States of America; 3 Forensic Biology/DNA, Denver Police Department Crime Laboratory, Denver, Colorado, United States of America; 4 Biostatistics Unit, Humanitas Clinical and Research Center, Rozzano, Milan, Italy; 5 Division of Pathology, Humanitas Clinical and Research Center, Rozzano, Milan, Italy; 6 University of Milan, Milan, Italy; 7 Division of Thoracic Surgery, Humanitas Clinical and Research Center, Rozzano, Milan, Italy; 8 Department of Radiotherapy, Humanitas Clinical and Research Center, Rozzano, Milan, Italy; 9 Lowe Center for Thoracic Oncology and Belfer Institute for Applied Cancer Science, Dana Farber Cancer Institute, Boston, Massachusetts, United States of America; University Medical Center Hamburg-Eppendorf, Germany

## Abstract

**Background:**

We aimed to investigate prevalence and prognostic role of *SOX2, PIK3CA, FGFR1* and *BRF2* gene gain in patients with surgically resected non-small cell lung cancer (NSCLC).

**Methods:**

*SOX2, PIK3CA, FGFR1* and *BRF2* gene copy number was assessed by fluorescence *in situ* hybridization (FISH) in arrayed tissue cores from 447 resected NSCLCs.

**Results:**

Increased gene copy number (FISH+) for *SOX2*, *PIK3CA*, *FGFR1* and *BRF2* was observed in 23.6%, 29.2%, 16.6% and 14.9% of cases, respectively. FISH+ status for each gene was significantly associated with smoking history, squamous cell carcinoma (SCC) histology, and increased copy number of the other studied genes. Multivariate analysis of overall survival indicated increased *SOX2* gene copy number (*P* = 0.008), stage I-II (*P*<0.001), and adenocarcinoma or SCC histology (*P* = 0.016) as independent, favorable prognostic factors. A statistically significant interaction was observed between stage and *SOX2* gene status (*P* = 0.021), indicating that the prognostic impact of *SOX2* gene gain differs across stages and is limited to patients with stage I-II disease (HR 0.44, 95% CI: 0.25–0.77; *P* = 0.004, adjusted for histology).

**Conclusions:**

Increased SOX2 gene copy number is an independent and favorable prognostic factor in surgically resected, early stage NSCLC, regardless of histology. SOX2, PIK3CA, FGFR1 and BRF2 gene gains are likely to occur concurrently, with potentially relevant implications for the development of new therapeutic strategies.

## Introduction

Lung cancer is the leading cause of cancer death worldwide and survival rates are poor. In the last few years, progress in the knowledge of lung cancer biology led to the identification of activated oncogenes that can be therapeutically targeted by novel agents. Among those are the tyrosine kinase inhibitors, such as gefitinib and erlotinib for patients with Epidermal Growth Factor Receptor (*EGFR*) mutations [1]–[2] and, more recently, crizotinib for subjects with Anaplastic Lymphoma Kinase (*ALK*) rearrangements [3]. These agents have significantly improved the outcome of biologically selected non-small cell lung cancer (NSCLC) patients with advanced disease, encouraging the identification of novel therapeutic targets.

The SRY (sex determining region Y)-box 2 (*SOX2*) gene, located on chromosome 3q26.33, encodes for a member of the SRY-related HMG-box family of transcription factors and has been implicated in pluripotency regulation in embryonic stem cells [4]. Upregulation of *SOX2* induces cell proliferation and anchorage-independent growth in lung squamous cell carcinoma (SCC) cell lines and drives lung tumorigenesis in mice [5]-[6]. Amplification of *SOX2* has been associated with lung SCC [5]–[8], with retrospective data suggesting a trend for improved survival in favor of *SOX2* gene gain in surgically resected patients [7]–[8].

The *PIK3CA* gene encodes for the catalytic subunit p110α of class IA phosphatidylinositol 3-kinases (PI3K) and has been identified to function as an oncogene in human malignancies activated by gene amplification or mutations [9]. Importantly, preclinical models showed that inhibition of the PI3K pathway impairs survival of NSCLC cells harboring *PIK3CA* gene alterations [10]–[11], and numerous novel PI3K, Akt and mTOR inhibitors have entered clinical trial testing for the treatment of PI3K-addicted cancers. *PIK3CA* is located on chromosome 3q26.32, close to the *SOX2* locus. Comparative genomic hybridization studies have shown distinct amplification levels for *SOX2* and *PIK3CA* in lung SCC with 3q26 gain [5], suggesting that they might play independent oncogenic roles.

The fibroblast growth factor receptor 1 (FGFR1), which is encoded by a gene located on chromosome 8p12.1, is a member of a four tyrosine kinase receptor family (FGFR1–4) and has been widely studied as a key receptor involved in embryonic development [12]. Amplification of *FGFR1* has been recently reported in up to 22% of lung SCC [13] and has been associated with sensitivity to FGFR1 tyrosine kinase inhibitors in multiple preclinical models, including lung SCC cell lines [13]–[15], leading to clinical trials of these agents in *FGFR1*-amplified tumors.

The B-related factor 2 (*BRF2*) gene, located on chromosome 8p11.23 very close to *FGFR1*, encodes a subunit of RNA polymerase III (Pol III) transcription initiation complex, which is responsible for the transcription of a number of noncoding RNA genes whose products are involved in protein synthesis, RNA processing and transcription [16]. The RNA pol III is often deregulated in cancer, and *BRF2* was recently identified as a novel oncogene in lung SCC activated by increased copy number [17]. In fact, *BRF2* amplification determines an increased RNA Pol III activity and sustained cell proliferation and survival *in vitro* [17].

Alterations in copy number of the *SOX2*, *PIK3CA*, *FGFR1* and *BRF2* genes have been individually studied in a number of NSCLC populations, particularly in lung SCC, with limited and sometimes conflicting data regarding their prognostic impact. Moreover, the prevalence of copy number gains of more than one gene and its effect on patient survival is still largely unexplored. The present study was conducted to investigate the prevalence of *SOX2, PIK3CA, FGFR1* and *BRF2* gene copy number changes in a large, unselected cohort of surgically resected NSCLC patients, to verify concurrent genomic gains, and to determine whether copy number alterations in these genes affect patient outcome.

## Patients and Methods

### Ethics Statement

Institutional Review Board (IRB) approval (#304/12) was obtained from the Istituto Clinico Humanitas ethical committee. Written informed consent was obtained from patients undergoing active follow up at our institution. IRB waived the requirements for written informed consent from patients that could not be reached. The study was conducted in accordance with ethical principles stated in the most recent version of the Declaration of Helsinki.

### Patients

This retrospective study was conducted in a previously reported cohort of 447 Caucasian NSCLC patients that received a radical resection of a primary NSCLC between 2000 and 2004 at Istituto Clinico Humanitas (Rozzano, Italy) [18]. A tissue microarray (TMA) was constructed using 0.6 mm diameter cores.

### Fluorescence *In Situ* Hybridization (FISH) Analysis

**Figure 1:**
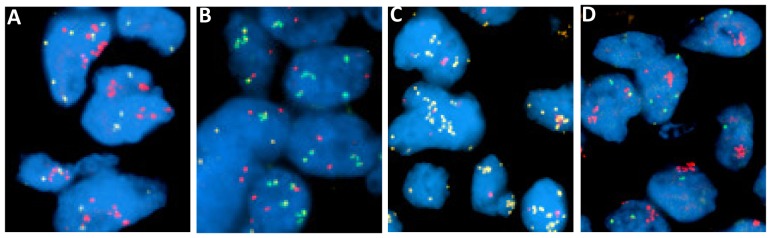
Specimen B5b showing gene amplification for *FGFR1* (A, red) and *BRF2* (B, green) and low copy number for *SOX2* (A, gold), and *PIK3CA* (B, red); specimen C4d showing gene amplification for *SOX2* (C, gold) and *PIK3CA* (D, red) and low copy number for *FGFR1* (C, red) and *BRF2* (D, green).

The Bacterial Artificial Chromosome (BAC) clones RP11-459K06, RP11-245C23, RP11-168H08 and RP11-350N15, respectively containing human DNA inserts from regions homologous to *SOX2*, *PIK3CA*, *BFR2* and *FGFR1*, were used for the enumeration FISH probes, according to previously described protocols [19]–[20]. Copy number per cell for each gene was enumerated on at least 50 tumor cells from 2 tissue cores per patient by an expert cytogeneticist blinded to patient data. In absence of validated FISH scoring criteria for these genes, a pre-specified cut-off of mean ≥4 gene copies/cell, previously used to define gene gain with other techniques [13], or presence of gene clusters indicating true gene amplification, was set to identify cases with increased gene copy number (FISH+). Examples of FISH patterns are shown in figure 1.

### Statistical Analysis

Associations between demographical and clinical characteristics and FISH status were estimated using the continuity adjusted Chi-square or the Fisher exact test, when appropriate. Pearson correlation coefficient was used to test the correlation between FISH status of each gene.

Overall survival (OS), calculated from the time of diagnosis to patient death or last contact, was evaluated using the Kaplan–Meier method. Differences between groups were evaluated by using log rank test. Hazard ratios with their corresponding 95% confidence intervals (95% CI) were calculated using the Cox proportional hazards regression model. Statistical significance was set at <0.05 for each analysis. All statistical analyses were carried out using R package.

## Results

### Patient Characteristics

The study included a cohort of 447 patients with surgically resected NSCLC. Details of the cohort have been previously described [18] and patient characteristics are summarized in table 1. Briefly, the majority of patients were male (82.8%), former (53.2%) or current smokers (36.0%), with histological grade 1 or 2 tumors (64.2%). All patients underwent radical surgery and tumors were staged according to the TNM classification with evidence of pathological stage I in 37.1% of cases, stage II in 22.1%, stage III in 32.7% and stage IV in 8.1%. The latter group included patients with a solitary metastatic lesion, mostly in the brain or adrenal glands, amenable of surgery or stereotactic radiosurgery. Patients with stage III pN2 disease (n = 101) received mediastinal post-operative radiotherapy. Overall, given the surgical timeframe (2000–2004), no patients with stage I-II tumors received post-operative systemic treatment, while only 18 subjects with stage III disease were treated with adjuvant platinum-based chemotherapy. The majority of patients were diagnosed with adenocarcinoma (54.6%), while 138 (30.9%) and 65 (14.5%) patients had SCC or other histology, respectively. After a median follow up of 60.4 months, 244 deaths occurred and median survival was 42.5 months. Significantly longer survival was observed for patients with stage I–II (herein referred to as “early stage”) tumors when compared with those with stage III–IV disease (*P*<0.001). Patients with SCC or adenocarcinoma had similar outcome and lived longer than patients with other histologies (*P*<0.001). No survival difference was observed according to gender, smoking history or pathological grade.

**Table 1:** Patient characteristics.

Characteristics	n	%^a^
**Total**	447	100
**Sex**		
Male	370	82.8
Female	77	17.2
**Smoking history**		
Never	40	8.9
Former	238	53.2
Current	161	36.0
Unknown	8	1.8
**Histology**		
Adenocarcinoma	244	54.6
SCC	138	30.9
Other	65	14.5
**Stage**		
I	166	37.1
II	99	22.1
III	146	32.7
IV	36	8.1
**Grade**		
I	32	7.2
II	255	57.0
III	158	35.3
Not reported	2	0.4

a percentages have been rounded and may not total 100%.

Abbreviation: SCC = squamous cell carcinoma.

### *SOX2* and *PIK3CA* Copy Number Gain

*SOX2* and *PIK3CA* gene status was successfully determined in 445 (99.6%) and 435 (97.3%) patients, respectively. Overall, *SOX2* increased gene copy number was detected in 105 cases (23.6%), with true gene amplification observed in 19 patients (4.3%). *PIK3CA* gene gain was found in 29.2% of patients, including 21 cases (4.8%) with true gene amplification. As expected given their physical proximity, a statistically significant association was observed for copy number gain between *SOX2* and *PIK3CA* (Pearson correlation coefficient: 0.78, *P*<0.001), with discordant FISH results between the two genes in only 9.9% of cases. This association was maintained across all histological subtypes (data not shown). As summarized in table 2, both *SOX2*+ and *PIK3CA*+ status significantly associated with male gender, former/current smoking history, and SCC histology. Additionally, *PIK3CA* gene gain was more commonly observed in grade III tumors (*P* = 0.040). All cases with *SOX2* true gene amplification were SCC, for an overall prevalence of 13.8% *SOX2*-amplified tumors in patients with SCC histology. Similarly, *PIK3CA* true gene amplification was found in 12.8% of cases with SCC, while only 4 patients with non-squamous tumors were *PIK3CA*-amplified, including 3 adenocarcinomas and one pleomorphic carcinoma.

**Table 2:** Associations between FISH status and patient characteristics.

	*SOX2*					*PIK3CA*					*FGFR1*					*BRF2*				
	(N = 445)					(N = 435)					(N = 445)					(N = 435)				
	−		+			−		+			−		+			−		+		
	n	%	n	%	*P value*	n	%	n	%	*P value*	n	%	n	%	*P value*	n	%	n	%	*P value*
**Total**	340	76.4	105	23.6		308	70.8	127	29.2		371	83.4	74	16.6		370	85.1	65	14.9	
**Sex**																				
Male	273	74.0	96	26.0	0.012	243	67.7	116	32.3	0.003	305	82.7	64	17.3	0.469	300	83.6	59	16.4	0.085
Female	67	88.2	9	11.8		65	85.5	11	14.5		66	86.8	10	13.2		70	92.1	6	7.9	
**Smoking History**																				
Never	37	92.5	3	7.5	0.010	35	89.7	4	10.3	0.005	37	92.5	3	7.5	0.121	38	97.4	1	2.6	0.018
Former + Current	296	74.4	102	25.6		265	68.3	123	31.7		327	82.2	71	17.8		324	83.5	64	16.5	
**Histology**																				
SCC	69	50.0	69	50.0	<0.001^a^	55	41.3	78	58.7	<0.001^a^	99	71.7	39	28.3	<0.001^a^	95	71.4	38	28.6	<0.001^a^
Adenocarcinoma	217	89.3	26	10.7		199	84.0	38	16.0		215	88.5	28	11.5		215	90.3	23	9.7	
Other	54	84.4	10	15.6		54	83.1	11	16.9		57	89.1	7	10.9		60	93.8	4	6.2	
**Stage**																				
I–II	200	75.5	65	24.5	0.654	179	69.4	79	30.6	0.495	222	83.8	43	16.2	0.883	221	85.7	37	14.3	0.773
III–IV	140	77.8	40	22.2		129	72.9	48	27.1		149	82.8	31	17.2		149	84.2	28	15.8	
**Grade**																				
I–II	226	79.0	60	21.0	0.089	207	74.2	72	25.8	0.040	241	84.3	45	15.7	0.545	239	85.4	41	14.6	0.881
III	112	71.3	45	28.7		99	64.3	55	35.7		128	81.5	29	18.5		129	84.3	24	15.7	

a SCC vs non-squamous histology.

Abbreviation: SCC = squamous cell carcinoma.

**Figure 2:**
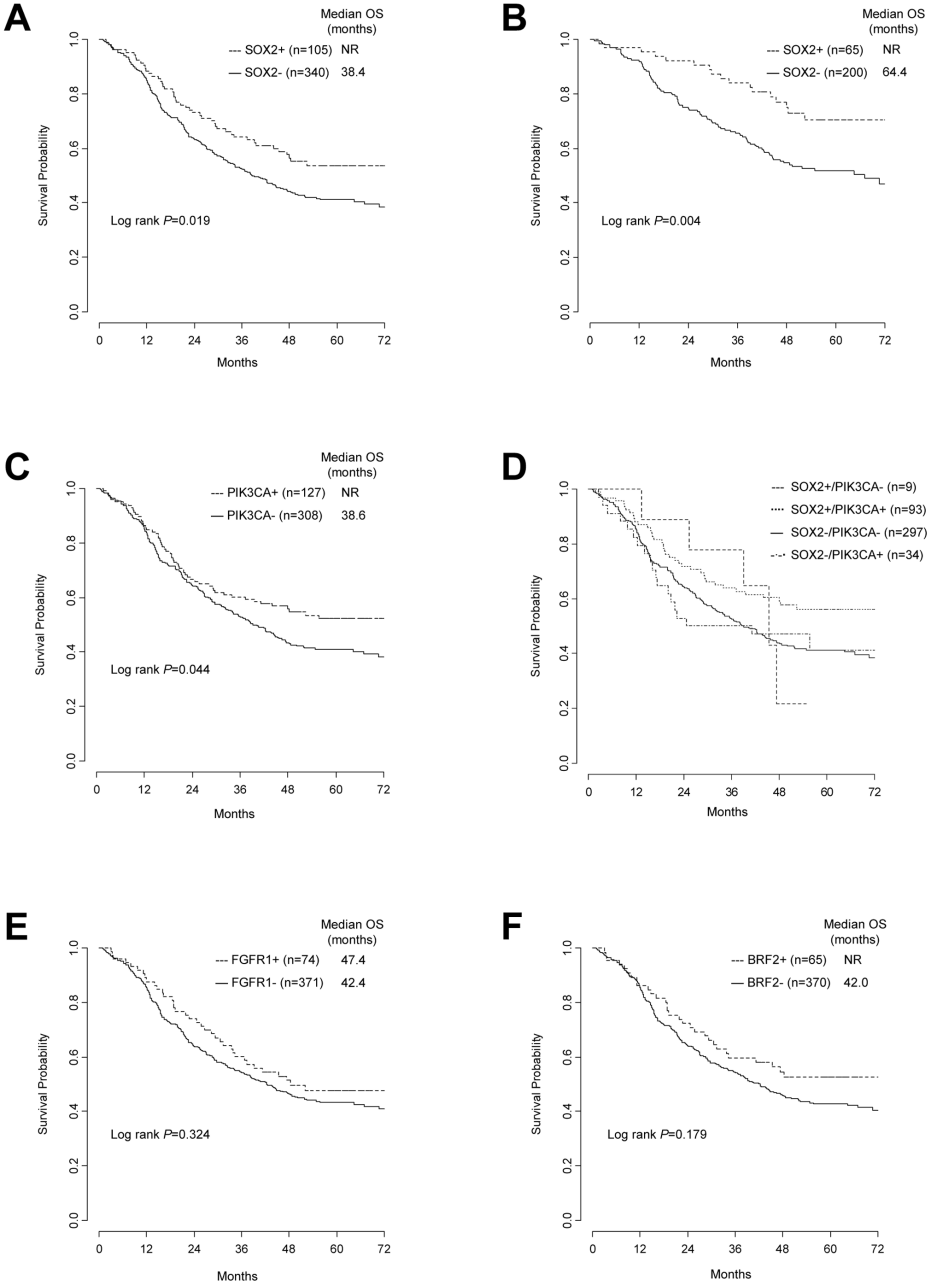
Kaplan-Meier overall survival estimates according to gene copy number. (A) *SOX2*, whole cohort. (B) *SOX2*, stage I-II. (C) *PIK3CA*, whole cohort. (D) *SOX2/PIK3CA*, whole cohort. (E) *FGFR1*, whole cohort. (F) *BRF2*, whole cohort. Abbreviations: OS = overall survival; NR = not reached.

*SOX2*+ patients had a significantly longer survival than *SOX2*- subjects (53.7% vs 41.1% 5-year OS, *P* = 0.019) (figure 2A), with no difference in outcome between cases with ≥4 mean gene copies/cell and those with true gene amplification (*P* = 0.937). Subgroup analyses according to single clinico-pathological characteristics, including stage, histology, sex, smoking status and grade, detected a significant survival advantage for *SOX2*+ patients in the subset of early stage (70.4% vs 51.8% 5-year OS, *P* = 0.004, figure 2B) and grade 3 tumors (52.3% vs 37.4% 5-year OS, *P* = 0.037). In patients with stage III–IV disease, no survival difference was detected between *SOX2*+ and *SOX2*- cases (*P* = 0.993), while a trend for a better outcome in favor of *SOX2*+ subjects was observed in the SCC subgroup (*P* = 0.053).

Survival data according to *PIK3CA* gene status were similar to *SOX2*, likely as a result of the substantial overlap between the FISH status of these two genes. In fact, a statistically significant survival advantage was observed for *PIK3CA*+ patients when compared with *PIK3CA*- patients in the whole population (52.2% vs. 40.9% 5-year OS, *P* = 0.044) (figure 2C). The survival advantage for *PIK3CA*+ patients remained statistically significant only in the subset of early stage tumors (69.5% vs 51.5% 5-year OS, *P* = 0.005), with a trend for improved survival in patients with SCC (*P* = 0.064).

We further aimed to explore the survival patterns of patients with discordant *SOX2/PIK3CA* gene status (n = 43). As shown in figure 2D, there was similar outcome between *SOX2-/PIK3CA*+ (n = 34) and double negative (n = 297) patients, while survival of *SOX2+/PIK3CA*- individuals (n = 9) appeared to be similar to that of the double positive group (n = 93).Those findings suggested that *PIK3CA* gene gain was not *per se* prognostically relevant.

### *FGFR1* and *BRF2* Copy Number Gain

A total of 445 (99.6%) patients were assessable for *FGFR1* gene copy number, with 16.6% FISH+ patients, including 37 cases (8.3%) with true gene amplification. *FGFR1* gene gain was significantly more common in patients with SCC than in those with adenocarcinoma or other histologies (SCC vs non-SCC: 28.3% vs 11.4%, *P*<0.001,) while no association with gender, smoking status, stage or grade was found (table 2). *FGFR1* true gene amplification occurred in 17.4% of SCC but was also observed in 3/26 (11.5%) tumors with neuroendocrine differentiation and 2/28 (7.1%) never-smokers with lung adenocarcinoma, including a female with a concurrent *EGFR* L858R mutation.

*BRF2* gene status was evaluated by FISH in 435 (97.3%) patients and increased gene copy number was observed in 14.9% of cases, including 27 patients (6.2%) with gene amplification. As shown in table 2, former/current smoking history and SCC histology were significantly associated with *BRF2*+ status. Likely in consequence of their close physical location on chromosome 8p11-12, a significant association was observed between *FGFR1* and *BRF2* gene copy number (Pearson correlation coefficient: 0.78, *P*<0.001), with only 5.3% of cases with discordant FISH results. The association between the two genes was independent of histology (data not shown). Furthermore, significant associations were observed between copy number gains of genes located on different chromosomes (*FGFR1* or *BRF2* with *SOX2* or *PIK3CA*, *P*<0.001 for each association), regardless of histology. Particularly, *SOX2* and *PIK3CA* gene gains were observed in 55.4% and 75.7%, respectively, of *FGFR1*+ tumors.

The *FGFR1*+ or *BRF2*+ patients survived longer than those with no or low copy number gain, although this difference did not reach statistical significance (*FGFR1*, *P* = 0.324; *BRF2*, *P* = 0.179) (figures 2E and 2F). *FGFR1* gene copy number failed to show a significant prognostic impact when analysing survival according to clinico-pathological characteristics, including sex, smoking status, stage, histology and grade (data not shown). Conversely, a significant survival advantage was observed for *BRF2*+ patients with early stage disease when compared with the *BRF2*- group (74.6% vs 54.0% 5-year OS, *P* = 0.040), while no prognostic relevance was observed for *BRF2* gene copy number in other patient subsets (data not shown). Due to the substantial overlap between *FGFR1* and *BRF2* FISH status, no survival differences were found in the whole population when grouping patients according to both genes (data not shown). Similarly, no survival impact was observed when combining *FGFR1* or *BRF2* with *SOX2* or *PIK3CA* FISH results in the whole population or in clinically-defined subgroups (data not shown).

### Multivariate Analysis

As previously mentioned, factors associated with survival were stage, histology, *SOX2* and *PIK3CA* gene copy number. Considering the strong association between *SOX2* and *PIK3CA* gene status, the two genes were assessed in two separate models. In the *SOX2* model, *SOX2* gene gain, early stage and adenocarcinoma/SCC histology emerged as independent prognostic factors for improved survival (HR_SOX2+ vs SOX2-_ 0.48, 95% CI: 0.28–0.83, *P* = 0.008; HR_stage I-II vs III-IV_ 0.49, 95% CI: 0.37–0.65; *P*<0.001; HR_ adenocarcinoma/SCC vs other_ 0.67, 95% CI: 0.46-0.93; *P* = 0.016). Importantly, a statistically significant interaction was observed between stage and *SOX2* gene status (*P* = 0.021), indicating that the prognostic impact of increased *SOX2* gene copy number differed according to tumor stage and was limited to patients with early stage disease (HR 0.44, 95% CI: 0.25–0.77; *P* = 0.004, adjusted for histology). In contrast, in the multivariate model considering *PIK3CA* gene copy number, stage and histology, *PIK3CA* gene gain failed to predict a significant longer survival (*P* = 0.135).

The survival impact observed for *BRF2* gene gain in patients with early stage disease was not confirmed in the multivariate model after correction for histology (*P* = 0.135), although the small number of *BRF2*+ cases precluded robust conclusions.

## Discussion

This study evaluated the prognostic relevance of copy number alterations of four oncogenes previously associated with lung SCC - *SOX2, PIK3CA, FGFR1* and *BRF2* - in a large cohort of surgically resected NSCLC patients. For the first time, we report increased *SOX2* gene copy number assessed by FISH as an independent favorable prognostic factor in patients with stage I and II NSCLC, regardless of histology.

SOX2, a transcription factor that plays a key role in embryonic development, has recently emerged as an attractive therapeutic target in lung cancer. In fact, amplification and/or overexpression of *SOX2* have been reported in lung SCC, lung adenocarcinoma and, more recently, small-cell lung cancer [5], [20]–[21]. Preclinical reports have been consistent in showing that SOX2 silencing leads to significant impairment of cell growth in lung cancer models, supporting SOX2 inhibition as a promising anticancer strategy in lung malignancies [5], [22].

Defining FISH+ status as mean ≥4 gene copies/cell or presence gene clusters (true gene amplification), *SOX2* gene gain was observed in about one fourth of NSCLCs and was significantly associated with male gender, exposure to tobacco smoke and SCC histology, as previously reported by others [5]–[7]. Particularly, half of lung SCC categorized as FISH+, with true gene amplification occurring in 13.8% of cases, while lung adenocarcinomas presented increased *SOX2* gene copy number in 10.7% of samples with no cases harbouring true gene amplification. These findings are roughly in line with other FISH studies in NSCLC, in which different scoring criteria were used [7]–[8].

The prognostic role of SOX2 has been investigated in retrospective NSCLC series. Despite data from a recent meta-analysis suggesting a favorable prognostic impact for SOX2 expression in NSCLC regardless of histology,[23] most studies showed that SOX2 protein overexpression predicted prolonged survival in surgically resected lung SCC [7]–[8], while its survival impact in lung adenocarcinoma remains controversial [21], [24]. Two studies have shown a significant association between SOX2 protein expression evaluated by immunohistochemistry and *SOX2* gene gain assessed by FISH, and suggested a favorable prognostic role for increased *SOX2* gene copy number in lung SCC, although statistical significance was not reached [7]–[8]. Particularly, Wilbertz et al. detected a trend for improved survival in lung SCC patients with ≥10 *SOX2* copies/cell compared with tumors with no or low level amplification, but this effect was lost in the multivariate model [7].

For the first time, we reported a statistically significantly improved overall survival for *SOX2* FISH+ patients with stage I and II NSCLC. Importantly, the favorable prognostic effect of *SOX2* gene gain observed in our study, where non-SCC patients accounted for about 38% of *SOX2*+ cases, was independent of histology and *PIK3CA* gene gain. Our findings contrast with those of another study where a negative survival impact for *SOX2* low level amplification, compared to lack of amplification, was observed in lung adenocarcinoma [7]. Additionally, our data indicate that the survival benefit observed in unselected early stage NSCLC was not limited to patients with true gene amplification, but was also extended to those with mean ≥4 *SOX2*copies/cell and no gene clusters. The discrepancies between our study and that of Wilbertz et al. might be explained by differences in FISH scoring criteria and patient clinical characteristics. The reason for the lack of a survival impact for *SOX2* gene gain in patients with stage III and IV disease in our cohort is unclear and should be further investigated. We speculate that *SOX2* gene gain is an early event in lung tumorigenesis and that tumor progression leads to additional molecular abnormalities that affect patient outcome.

FGFR1 has recently emerged as a promising target in NSCLC after the gene was reported as amplified in about 20% of lung SCC [13]–[14], [25], leading to early phase clinical trials of anti-FGFR1 agents in *FGFR1*-amplified NSCLC. Our data confirmed the previously reported association between increased *FGFR1* gene copy number and SCC histology. Importantly, *FGFR1* gene gain was also found in 11.4% of non-squamous tumors, including true gene amplification in about 7% of adenocarcinoma patients with never smoking history - the same population with an increased likelihood of harboring *EGFR* mutations or *ALK* rearrangements - and 11% among neuroendocrine tumors. Although the small numbers of these subgroups preclude any firm conclusion, our data suggest that *FGFR1* gene copy number assessment should be pursued in selected patients with non-squamous tumors to identify candidates for anti-FGFR1 agents.

In the present study, we observed that concurrent copy number gains of *SOX2, PIK3CA, FGFR1* or *BRF2* were common. While the strong correlation between *SOX2* and *PIK3CA* and between *FGFR1* and *BRF2* gene status was anticipated because of their proximity on chromosomes 3q26 and 8p11-12, respectively, our study is the first to report statistically significant associations between copy number gains in both genomic regions, regardless of histology. In contrast, Weiss et al. described that *FGFR1* and *SOX2* amplifications were mutually exclusive in lung SCC, although in that study the genomic gain was investigated by single-nucleotide polymorphism arrays and different thresholds for copy number changes were used [13]. Our findings should raise caution in the development of PI3K or FGFR1 inhibitors as single agents in NSCLCs with *PIK3CA* or *FGFR1* gene gain, respectively. In fact, in presence of concurrent genomic gain of multiple oncogenes concomitant inhibition of more than one target could be required to effectively impair tumor growth. This concept is supported by the observation that PI3K inactivation with RNA interference technology, in lung SCC cell lines with 3q26 amplification, produced only limited effects on cell proliferation as opposed to *SOX2* knockdown [5]. Moreover, an intriguing observation was the coexistence of *FGFR1* true gene amplification and *EGFR* L858R mutation in a female, never smoker patient with lung adenocarcinoma, suggesting that some NSCLCs could be co-dependent on FGFR1 and EGFR for survival. Overall, these data suggest that in some tumors the assessment of a single drug target may not be sufficient to predict drug sensitivity, addressing the need for a deeper understanding of NSCLC biology.

Our study showed no statistically significant prognostic role for *FGFR1* or *BRF2* gene copy number in surgically resected NSCLC patients when using our predefined FISH scoring criteria, although a trend for improved survival for *FGFR1*+ or *BRF2*+ patients could be observed in the whole population. Other investigators have recently explored whether *FGFR1* gene gain affects survival in NSCLC with inconclusive results [13], [26]–[29]. In fact, while some authors reported a survival advantage for NSCLCs with increased *FGFR1* gene copy number [28], a recent Korean study showed that *FGFR1* high level amplification negatively affects survival in surgically resected lung SCC [27]. Different techniques, scoring methods, and criteria for patient selection (i.e. histology, ethnicity, stage, perioperative treatments) might account for the differences observed across studies, encouraging the assessment of the prognostic role of *FGFR1* gene copy number in uniformly selected cohorts.

In conclusion, we showed for the first time that increased *SOX2* gene copy number is significantly associated with improved survival in surgically resected stage I and II NSCLC patients, regardless of histology. We also found that *SOX2* gene gain is associated with copy number gains of other actionable oncogenes, including *FGFR1* and *PIK3CA*. Our findings confirm the complexity of NSCLC biology and encourage the exploration of novel therapeutic combinations.

## References

[1] Fukuoka M, Wu YL, Thongprasert S, Sunpaweravong P, Leong SS, et al (2011) Biomarker analyses and final overall survival results from a phase III, randomized, open-label, first-line study of gefitinib versus carboplatin/paclitaxel in clinically selected patients with advanced non-small-cell lung cancer in Asia (IPASS). J Clin Oncol 29: 2866–2874. 21670455 10.1200/JCO.2010.33.4235

[2] Rosell R, Carcereny E, Gervais R, Vergnenegre A, Massuti B, et al (2012) Erlotinib versus standard chemotherapy as first-line treatment for European patients with advanced EGFR mutation-positive non-small-cell lung cancer (EURTAC): a multicentre, open-label, randomised phase 3 trial. Lancet Oncol 13: 239–246. 22285168 10.1016/S1470-2045(11)70393-X

[3] Kwak EL, Bang YJ, Camidge DR, Shaw AT, Solomon B, et al (2010) Anaplastic lymphoma kinase inhibition in non-small-cell lung cancer. N Engl J Med 363: 1693–1703. 20979469 10.1056/NEJMoa1006448 PMC3014291

[4] Kamachi Y, Uchikawa M, Kondoh H (2000) Pairing SOX off: with partners in the regulation of embryonic development. Trends Genet 16: 182–187. 10729834 10.1016/s0168-9525(99)01955-1

[5] Bass AJ, Watanabe H, Mermel CH, Yu S, Perner S, et al (2009) SOX2 is an amplified lineage-survival oncogene in lung and esophageal squamous cell carcinomas. Nat Genet 41: 1238–1242. 19801978 10.1038/ng.465 PMC2783775

[6] Hussenet T, Dali S, Exinger J, Monga B, Jost B, et al (2010) SOX2 is an oncogene activated by recurrent 3q26.3 amplifications in human lung squamous cell carcinomas. PLoS One 5: e8960. 20126410 10.1371/journal.pone.0008960 PMC2813300

[7] Wilbertz T, Wagner P, Petersen K, Stiedl AC, Scheble VJ, et al (2011) SOX2 gene amplification and protein overexpression are associated with better outcome in squamous cell lung cancer. Mod Pathol 24: 944–953. 21460799 10.1038/modpathol.2011.49

[8] Brcic L, Sherer CK, Shuai Y, Hornick JL, Chirieac LR, et al (2012) Morphologic and clinicopathologic features of lung squamous cell carcinomas expressing Sox2. American journal of clinical pathology 138: 712–718. 23086772 10.1309/AJCP05TTWQTWNLTN

[9] Cantley LC (2002) The phosphoinositide 3-kinase pathway. Science 296: 1655–1657. 12040186 10.1126/science.296.5573.1655

[10] Yamamoto H, Shigematsu H, Nomura M, Lockwood WW, Sato M, et al (2008) PIK3CA mutations and copy number gains in human lung cancers. Cancer Res 68: 6913–6921. 18757405 10.1158/0008-5472.CAN-07-5084 PMC2874836

[11] Spoerke JM, O'Brien C, Huw L, Koeppen H, Fridlyand J, et al (2012) Phosphoinositide 3-Kinase (PI3K) Pathway Alterations Are Associated with Histologic Subtypes and Are Predictive of Sensitivity to PI3K Inhibitors in Lung Cancer Preclinical Models. Clin Cancer Res 18: 6771–6783. 23136191 10.1158/1078-0432.CCR-12-2347

[12] Eswarakumar VP, Lax I, Schlessinger J (2005) Cellular signaling by fibroblast growth factor receptors. Cytokine Growth Factor Rev 16: 139–149. 15863030 10.1016/j.cytogfr.2005.01.001

[13] Weiss J, Sos ML, Seidel D, Peifer M, Zander T, et al (2010) Frequent and focal FGFR1 amplification associates with therapeutically tractable FGFR1 dependency in squamous cell lung cancer. Sci Transl Med 2: 62ra93. 10.1126/scitranslmed.3001451 PMC3990281 21160078

[14] Dutt A, Ramos AH, Hammerman PS, Mermel C, Cho J, et al (2011) Inhibitor-sensitive FGFR1 amplification in human non-small cell lung cancer. PLoS One 6: e20351. 21666749 10.1371/journal.pone.0020351 PMC3110189

[15] Zhang J, Zhang L, Su X, Li M, Xie L, et al (2012) Translating the Therapeutic Potential of AZD4547 in FGFR1-Amplified Non-Small Cell Lung Cancer through the Use of Patient-Derived Tumor Xenograft Models. Clin Cancer Res 18: 6658–6667. 23082000 10.1158/1078-0432.CCR-12-2694

[16] Cabarcas S, Schramm L (2011) RNA polymerase III transcription in cancer: the BRF2 connection. Mol Cancer 10: 47. 21518452 10.1186/1476-4598-10-47 PMC3098206

[17] Lockwood WW, Chari R, Coe BP, Thu KL, Garnis C, et al (2010) Integrative genomic analyses identify BRF2 as a novel lineage-specific oncogene in lung squamous cell carcinoma. PLoS Med 7: e1000315. 20668658 10.1371/journal.pmed.1000315 PMC2910599

[18] Cappuzzo F, Marchetti A, Skokan M, Rossi E, Gajapathy S, et al (2009) Increased MET gene copy number negatively affects survival of surgically resected non-small-cell lung cancer patients. J Clin Oncol 27: 1667–1674. 19255323 10.1200/JCO.2008.19.1635 PMC3341799

[19] Arcaroli JJ, Quackenbush KS, Powell RW, Pitts TM, Spreafico A, et al (2012) Common PIK3CA mutants and a novel 3' UTR mutation are associated with increased sensitivity to saracatinib. Clin Cancer Res 18: 2704–2714. 22553375 10.1158/1078-0432.CCR-11-3167 PMC3836589

[20] Rudin CM, Durinck S, Stawiski EW, Poirier JT, Modrusan Z, et al (2012) Comprehensive genomic analysis identifies SOX2 as a frequently amplified gene in small-cell lung cancer. Nat Genet 44: 1111–1116. 22941189 10.1038/ng.2405 PMC3557461

[21] Sholl LM, Barletta JA, Yeap BY, Chirieac LR, Hornick JL (2010) Sox2 protein expression is an independent poor prognostic indicator in stage I lung adenocarcinoma. Am J Surg Pathol 34: 1193–1198. 20631605 10.1097/PAS.0b013e3181e5e024 PMC2923819

[22] Xiang R, Liao D, Cheng T, Zhou H, Shi Q, et al (2011) Downregulation of transcription factor SOX2 in cancer stem cells suppresses growth and metastasis of lung cancer. Br J Cancer 104: 1410–1417. 21468047 10.1038/bjc.2011.94 PMC3101944

[23] Chen Y, Huang Y, Chen J, Wang S, Zhou J (2013) The Prognostic Value of SOX2 Expression in Non-Small Cell Lung Cancer: A Meta-Analysis. PLoS One 8: e71140. 23990933 10.1371/journal.pone.0071140 PMC3747201

[24] Velcheti V, Schalper K, Yao X, Cheng H, Kocoglu M, et al (2013) High SOX2 levels predict better outcome in non-small cell lung carcinomas. PLoS One 8: e61427. 23620753 10.1371/journal.pone.0061427 PMC3631238

[25] Schildhaus HU, Heukamp LC, Merkelbach-Bruse S, Riesner K, Schmitz K, et al (2012) Definition of a fluorescence in-situ hybridization score identifies high- and low-level FGFR1 amplification types in squamous cell lung cancer. Mod Pathol 25: 1473–1480. 22684217 10.1038/modpathol.2012.102 PMC4089812

[26] Heist RS, Mino-Kenudson M, Sequist LV, Tammireddy S, Morrissey L, et al (2012) FGFR1 Amplification in Squamous Cell Carcinoma of The Lung. J Thorac Oncol 7: 1775–1780. 23154548 10.1097/JTO.0b013e31826aed28 PMC3500511

[27] Kim HR, Kim DJ, Kang DR, Lee JG, Lim SM, et al (2012) Fibroblast Growth Factor Receptor 1 Gene Amplification Is Associated With Poor Survival and Cigarette Smoking Dosage in Patients With Resected Squamous Cell Lung Cancer. J Clin Oncol 31: 731–737. 23182986 10.1200/JCO.2012.43.8622

[28] Tran TN, Selinger CI, Kohonen-Corish MR, McCaughan BC, Kennedy CW, et al (2013) Fibroblast growth factor receptor 1 (FGFR1) copy number is an independent prognostic factor in non-small cell lung cancer. Lung Cancer 81: 462–467. 23806793 10.1016/j.lungcan.2013.05.015

[29] Craddock KJ, Ludkovski O, Sykes J, Shepherd FA, Tsao MS (2013) Prognostic value of fibroblast growth factor receptor 1 gene locus amplification in resected lung squamous cell carcinoma. J Thorac Oncol 8: 1371–1377. 24077455 10.1097/JTO.0b013e3182a46fe9

